# LL-37 boosts immunosuppressive function of placenta-derived mesenchymal stromal cells

**DOI:** 10.1186/s13287-016-0448-3

**Published:** 2016-12-30

**Authors:** Martha Oliveira-Bravo, Bruno Braga Sangiorgi, Josiane Lilian dos Santos Schiavinato, Juliana Lott Carvalho, Dimas Tadeu Covas, Rodrigo Alexandre Panepucci, Francisco de Assis Rocha Neves, Octávio Luiz Franco, Rinaldo Wellerson Pereira, Felipe Saldanha-Araujo

**Affiliations:** 1 Laboratório de Farmacologia Molecular, Departamento de Ciências da Saúde, Universidade de Brasília, Campus Darcy Ribeiro, Brasília, DF Brazil; 2 Laboratório de Hematologia, Departamento de Clínica Médica, Universidade de São Paulo, Ribeirão Preto, SP Brazil; 3 Laboratório de Análises Proteômicas e Bioquímicas, Centro de Ciências Genômicas e Biotecnologia, Universidade Católica de Brasília, Brasília, DF Brazil; 4 S-Inova Biotech, Pós-graduação em Biotecnologia, Universidade Católica Dom Bosco, Campo Grande, MS Brazil

**Keywords:** Mesenchymal stromal cells, LL-37, TLR, GvHD

## Abstract

**Background:**

Although promising for graft-versus-host disease (GvHD) treatment, MSC therapy still faces important challenges. For instance, increasing MSC migratory capacity as well as potentializing immune response suppression are of interest. For GvHD management, preventing opportunistic infections is also a valuable strategy, since immunocompromised patients are easy targets for infections. LL-37 is a host defense peptide (HDP) that has been deeply investigated due to its immunomodulatory function. In this scenario, the combination of MSC and LL-37 may result in a robust combination to be clinically used.

**Methods:**

In the present study, the effects of LL-37 upon the proliferation and migratory capacity of human placenta-derived MSCs (pMSCs) were assessed by MTT and wound scratch assays. The influence of LL-37 over the immunosuppressive function of pMSCs was then investigated using CFSE cell division kit. Flow cytometry and real-time PCR were used to investigate the molecular mechanisms involved in the effects observed.

**Results:**

LL-37 had no detrimental effects over MSC proliferation and viability, as assessed by MTT assay. Moreover, the peptide promoted increased migratory behavior of pMSCs and enhanced their immunomodulatory function over activated human PBMCs. Strikingly, our data shows that LL-37 treatment leads to increased TLR3 levels, as shown by flow cytometry, and to an increased expression of factors classically related to immunosuppression, namely IDO, IL-10, TGF-β, IL-6, and IL-1β.

**Conclusions:**

Taken together, our observations may serve as groundwork for the development of new therapeutic strategies based on the combined use of LL-37 and MSCs, which may provide patients not only with an enhanced immunosuppression regime, but also with an agent to prevent opportunistic infections.

## Background

Mesenchymal stem cells (MSCs) have riveted the attention of the scientific community, due to their surprisingly effective ability to suppress immune response. Although not completely understood, such immunomodulatory function of MSCs occurs mainly by secretion of soluble factors, cell-cell contact, and induction of regulatory T cells [1]. Due to their immune response modulation properties, MSCs have been used in clinical settings, especially in order to treat situations where the immune response is exacerbated, as occurs in the graft-versus-host disease (GvHD), a common life-threatening disorder characterized by damage in the liver, skin, mucosa, and gastrointestinal tract of patients who received allogeneic hematopoietic cell transplantation [2]. In GvHD patients, harsh immunosuppressive regimens are performed to control the disease, though at a cost of higher frailty against infections. Thus, relapse, lethal GvHD, and opportunistic infections are the main causes of death of patients following transplantation [3].

Despite promising, the results obtained by MSC treatment of GvHD are still heterogeneous, and there is much room for improvement [4, 5]. Moreover, the cost and time required for expansion of MSCs prior to clinical use constitute an important limitation to a more widespread clinical application of MSCs [6]. Therefore, it is imperative to search for strategies aiming at boosting the immunosuppressive role of MSCs while facilitating their production and engraftment. In this sense, research focused on MSC licensing is currently underway, with some exciting results in mice, in which MSCs’ immunosuppresive functions are enhanced if cells are presented with interferon gamma (INF-ɣ) prior to GvHD treatment [7, 8]. However, the same observations were not reproduced when human MSCs were INF-γ licensed and used to control T cell proliferation in vitro [9] and to control immune response in vivo [10].

An additional strategy under investigation, aiming to enhance MSC-mediated effects is the modulation of Toll-like receptors (TLRs), since priming of MSCs with specific TLR agonists efficiently directs these cells to take an anti-inflammatory or pro-inflammatory phenotype, therefore importantly influencing MSCsʼ immunosuppressive potential [11, 12]. Less innovative, but still relatively successful, is the combination of MSC therapy with immunosuppressants. So far, achieved results are conflicting, being that some authors show benefits when following this formula, while others emphasize that immunosuppressants actually compromise MSC function and antagonize their immunomodulatory effects [13–16].

Currently, several host defense peptides (HDPs) have been investigated due to their immunomodulatory functions [17]. Although several classes of HDPs exist, cathelicidin LL-37 (LL-37) is the sole member of the cathelicidin family found in humans [18]. First described in leukocytes [19], LL-37 can in fact be found in various cell types, tissues, and body fluids, including bone marrow [20], saliva [21], amniotic fluid [22], lung epithelia [23], breast milk [24], and others. Despite being harmless to human cells in low concentrations, LL-37 exhibits clear antimicrobial activities against several opportunistic pathogens [18, 25], being capable of causing membrane disruption [26]. Interestingly, LL-37 production constitutes a mechanism by which MSCs exert their antimicrobial activity [27]. In human cells, it has been shown that LL-37 mediates important signaling by binding to the formyl peptide receptor-like 1 (FPRL1) [28], purinergic receptor P2X7 (P2X7) [29], and epidermal growth factor receptor (EGFR) [30]. LL-37 also demonstrated the capability of modulating pathways related to immune and inflammatory response, stimulating both pro-inflammatory and anti-inflammatory response, depending on the environmental and cellular context in which it is exposed [26]. Among described events lead by LL-37 chemoattraction of T cells, mononuclear cells, and neutrophils [31, 32]; antagonism of pro-inflammatory cytokine production [33, 34]; and induction of strong anti-inflammatory response through modulation of TLR signaling and stimulation of interleukin 10 (IL-10) production [35, 36] are of note.

Given the LL-37 protective effects against the opportunistic microorganisms and its broad-spectrum action on immune response modulation, here we evaluated if this peptide could exert any impact on the immunosuppressive function of human placental-derived MSCs (pMSCs). Our data shows that, when in contact with LL-37, MSCs preserve their viability and proliferation properties, but also induce higher suppression of T cell activation, measured as proliferation following mitogenic stimulation. This effect is not observed when MSCs are pretreated with LL-37, therefore not working as a licensing agent, but as an adjuvant. Nevertheless, LL-37 action is not related to any direct effect over T cells, since LL-37 actually enhances T cell proliferation in the absence of MSCs. Strikingly, LL-37 induced MSCs to express the anti-inflammatory factors indoleamine 2,3-dioxygenase (IDO), IL-10 and transforming growth factor beta (TGF-β) and lead to an increased TLR3 expression, which could explain, at least in part, our observations. To the best of our knowledge, this is the first demonstration that LL-37 in fact boosts human MSC immunosuppressive effect.

## Methods

### Isolation and culture

Placentas (n = 3) were collected from normal full-term pregnancies following informed consent, being the pMSCs obtained from cotyledons present toward the maternal side of the placenta. Following isolation, the cotyledons were washed with a solution of phosphate-buffered saline (PBS) containing 100 U penicillin/streptomycin and cut into small pieces. After, harvested pieces were submitted to enzymatic digestion by incubation with 0.5% collagenase type IA (Sigma-Aldrich, St. Louis, MO, USA) for 45 minutes. Then, the enzyme was inactivated by Roswell Park Memorial Institute (RPMI) medium supplemented with 5% fetal bovine serum (FBS; HyClone, Logan, UT, USA). The obtained homogenate was washed in PBS and suspended in basal medium, composed of alpha--minimum essential medium (α-MEM) supplemented with 15% fetal bovine serum (FBS; HyClone, Logan, UT, USA), 2 mM glutamine, and 100 U penicillin/streptomycin (Sigma-Aldrich, St. Louis, MO, USA). Cells were then plated and allowed to adhere overnight. Non-adherent cells were washed away during medium changes. pMSCs from the fourth to sixth passage were used for experiments. All experiments performed in this study were conducted with the approval of the Institutional Ethics Committee.

### Experimental design

Isolated pMSCs were submitted to different procedures, as follows. Untreated pMSCs (control) received no pretreatment or treatment. In this group, cells were plated and maintained in basal media during the whole experiment. Pretreated pMSCs were plated on day −2 and incubated with 1 or 10 μg/mL of LL-37 from day −2 to day 0. On day 0, LL-37 was washed out three times with PBS and cells were maintained in basal media from day 0 to the end of the experiment, being that no further treatment with LL-37 was performed in this group. Finally, treated pMSCs were plated on day −2 and maintained in basal media until day 0. On day 0, treated pMSCs received either 1 or 10 μg/mL of LL-37, which was maintained until the day of experiments.

### LL-37 synthesis and mass spectrometry analyses

The original sequence of LL-37 was purchased from Peptides 2.0 (Chantilly, VA, USA) and then re-suspended in 500 μl of ultrapure water with 95% purity degree. In order to remove impurities, LL-37 was applied onto a reversed-phase high-performance liquid chromatography (HPLC) column (Vydac C18 TP522; Hichrom, Reading, UK) and eluted with a linear acetonitrile gradient (5–50% for 15 min), at flow rate of 1 ml.min^−1^. Peptide elution was monitored at 216 nm. Purified peptide was checked by mass spectrometry (MALDI-TOF/TOF Ultraflex II; Bruker Daltonics, Bremen, Germany).

### pMSC characterization

5 × 10^5^ pMSCs per tube were phenotypically characterized by flow cytometry (FACSCalibur, BD Biosciences, Franklin Lakes, NJ, USA), using the following antibodies CD105-PerCP, CD54-PE, CD44-FITC, CD49e-PE, CD166-PE, CD13-APC, HLA-ABC-PE, CD45-FITC, CD14-PE, CD51,61-FITC, CD106-FITC, CD34-PerCP, CD31-FITC, and HLA-DR-FITC (Pharmigen, BD Biosciences, Franklin Lakes, NJ, USA). Ten thousand events were recorded for each sample and data was analyzed using CellQuest software (Becton Dickinson, Franklin Lakes, NJ, USA). In addition, pMSCs were functionally characterized by multipotent differentiation in adipocytes and osteocytes, as previously described [37].

### pMSC viability

The effect of LL-37 over pMSC growth (proliferation and/or viability) was assessed by measuring the 3-(4.5-dimethylthiazol-2-yl)-2,5-diphenyl tetrazolium bromide (MTT) dye absorbance of the cells. For this, we cultured 2 × 10^3^ pMSCs in the following conditions: untreated (control); pretreated (1 or 10 μg/mL of LL-37 treatment from day −2 to day 0) or treated (1 or 10 μg/mL of LL-37 was added in the 0 and kept until MTT assessment, which occurred at days 1, 3, 5, and 7). For the pretreatment group, LL-37 was washed out three times with PBS at day 0. MTT assay was performed at 1, 3, 5, and 7 days post plating. In these time points, 20 μL of MTT (5 mg/mL) was added in each well and the plates incubated for 3 h. After this period, MTT and medium were discarded and replaced by DMSO, and the plate was homogenized for 15 minutes. The optical density was read on a DTX 800 Series Multimode Detector (Beckman Coulter, Brea, CA, USA) at 570 nm.

### pMSCs migration

The effect of LL-37 on pMSCs migration was determined by wound scratch assay, according to Liang et al. [38]. This assay was performed following the same design used to evaluate the effects of LL-37 in pMSCs viability. In brief, 2 × 10^5^ pMSCs were seeded in 6-well plates and cultured until confluence (day −2). On day 0, serum deprivation conditions were imposed and the pMSC monolayers were gently scratched across the center of the well using a 200 μL pipette tip. Untreated pMSCs were kept in basal media during the whole experiment. Pretreated pMSCs were incubated with 1 or 10 μg/mL of LL-37 from day −2 to day 0. Treated pMSCs were incubated with 1 or 10 μg/mL of LL-37 from day 0 until the end of the experiment. Scratch zone was photographed at day 0 and at 48 h and 96 h using a Zeiss Primo Vert microscope equipped with a digital camera (Carl Zeiss, Heidelberg, Germany). The scratch was then measured using the ImageJ software (National Institutes of Health, Bethesda, MD, USA).

### Isolation and activation of peripheral blood mononuclear cells (PBMCs)

Peripheral blood mononuclear cells (PBMCs) were isolated from the blood of healthy volunteers by centrifugation using Ficoll-Paque PLUS (Amersham Biosciences, Uppsala, Sweden) and washed three times with PBS. Isolated PBMCs were then activated with 10 μg/mL phytohemagglutinin (PHA, Sigma-Aldrich, St. Louis, MO, USA) and used in co-culture experiments after being stained with 2.5 μM carboxyfluorescein succinimidyl ester (CFSE), as previously described [39].

### Immunosuppression assays

The effect of LL-37 over the immunosuppressive capacity of pMSCs was assessed in two conditions. First, we tested the effect of LL-37 as an adjuvant, used directly in the co-culture of pMSCs and CFSE-labeled, PHA-activated PBMCs. In this scenario, 3.5 × 10^4^ pMSCs and 3.5 × 10^5^ PHA-activated PBMCs (1:10 ratio) were co-cultured for 5 days, and 1 or 10 μg/mL LL-37 was added in the first or third day of the experiment. On the fifth day, PBMCs were collected and incubated with anti-CD3 (APC-conjugated; Invitrogen, Waltham, MA, USA) for T cell proliferation assay by flow cytometry. In the second condition, we tested the capacity of pMSCs pretreated with LL-37 to modulate the proliferation of CFSE-labeled T cells. For this, pMSCs (3.5 × 10^4^) were cultured with 1 or 10 μg/mL LL-37 for 2 days. After this period, the medium was discarded, pMSCs were washed three times with PBS and co-cultured with PHA-activated PBMCs (1:10 ratio) for 5 days. In parallel, the effect of LL-37 on the proliferation of PHA-activated PBMCs cultured alone was also investigated, as a control. On the fifth day of the assay, PBMCs were recovered, stained with anti-CD3, so T cell proliferation could be determined by flow cytometry.

### TLR expression on pMSC

TLR3 and TLR4 expression levels of pMSCs (3.5 × 10^4^) untreated and treated for 2 days with 1 μg/mL of LL-37 were investigated via flow cytometry using monoclonal antibodies (PE-conjugated; eBioscience, San Diego, CA, USA), as previously described [40]. The same fluorochrome-labeled isotype-matched monoclonal antibodies were used as controls. Intracellular antibody staining was achieved after fixation and permeabilization of the cells as indicated by the manufacturer (cytofix/cytoperm buffers, BD Biosciences, San Jose, CA, USA). Ten thousand events were recorded for each sample and data was analyzed using FlowJo software 10.0.7 (FlowJo LLC, Ashland, OR, USA).

### RNA isolation and real-time PCR

Gene expression analysis was performed in untreated pMSCs and pMSCs treated with 1 or 10 μg/mL LL-37 for 2 days. Cells were recovered and submitted to ribonucleic acid (RNA) extraction using the RNEasy Mini Kkit, as indicated by the manufacturer (Qiagen, Valencia, CA, USA). Briefly, after collection, 3 × 10^4^ pMSCs were disrupted in RLT buffer, ethanol was added and samples were transferred to the kitʼs spin column. Total RNA was retained in the column while contaminants were washed away using buffer RW1 and buffer RPE. Then, total RNA was eluted. RNA amount and quality were determined using NanoDrop 1000 spectrophotometer (NanoDrop, Wilmington, DE, USA) and analyzed by a Bioanalyzer (Agilent Genomics, Santa Clara, CA, USA). Samples with RIN 8 or higher were used for complementary DNA (cDNA) production. Total RNA was reverse transcribed using the High Capacity cDNA Archive Kit, and real-time PCR was performed using TaqMan probes and MasterMix (Applied BioSystems, Foster City, CA, USA), following manufacturer’s instructions.

PCR for epidermal growth factor receptor 1 (EGFR1) was performed using the sense primer 5′-GATACCCAGGACCCAG-3′ and the antisense primer 5′-GCGACAATGAAAAACT-3′. As a control, GAPDH was amplified using sense primer 5′-ACATCGCTCAGACACCATG-3′ and antisense primer 5′- TGTAGTTGAGGTCAATGAAGGG-3′.

Real-time PCR for tumor necrosis factor (TNF) (Hs01113624), TGF-β (Hs00998133), interleukin 6 (IL-6) (Hs00985639), IDO (Hs00984148), Galectin-1 (Hs00355202), interleukin 1 beta (IL-1β) (Hs00174097) and IL-10 (Hs00961622) was done in duplicate and the relative fold value obtained by the 2 –ΔΔCt method [41]. To normalize sample loading, the differences of threshold cycles (ΔCt) were obtained by subtracting the Ct value for the internal reference (β-actin) from the Ct values of the evaluated genes. The median Ct values of the samples from untreated pMSCs were used as a reference.

### Statistical analysis

The results were given as mean ± SEM of independent experiments. Statistical analysis and graph generation were performed using Prism 5 software (GraphPad Software Inc., San Diego, CA, USA). Statistical significance was calculated using *t* test analyses. The value *p* < 0.05 was considered statistically significant.

## Results

### pMSC characterization

**Fig. 1:**
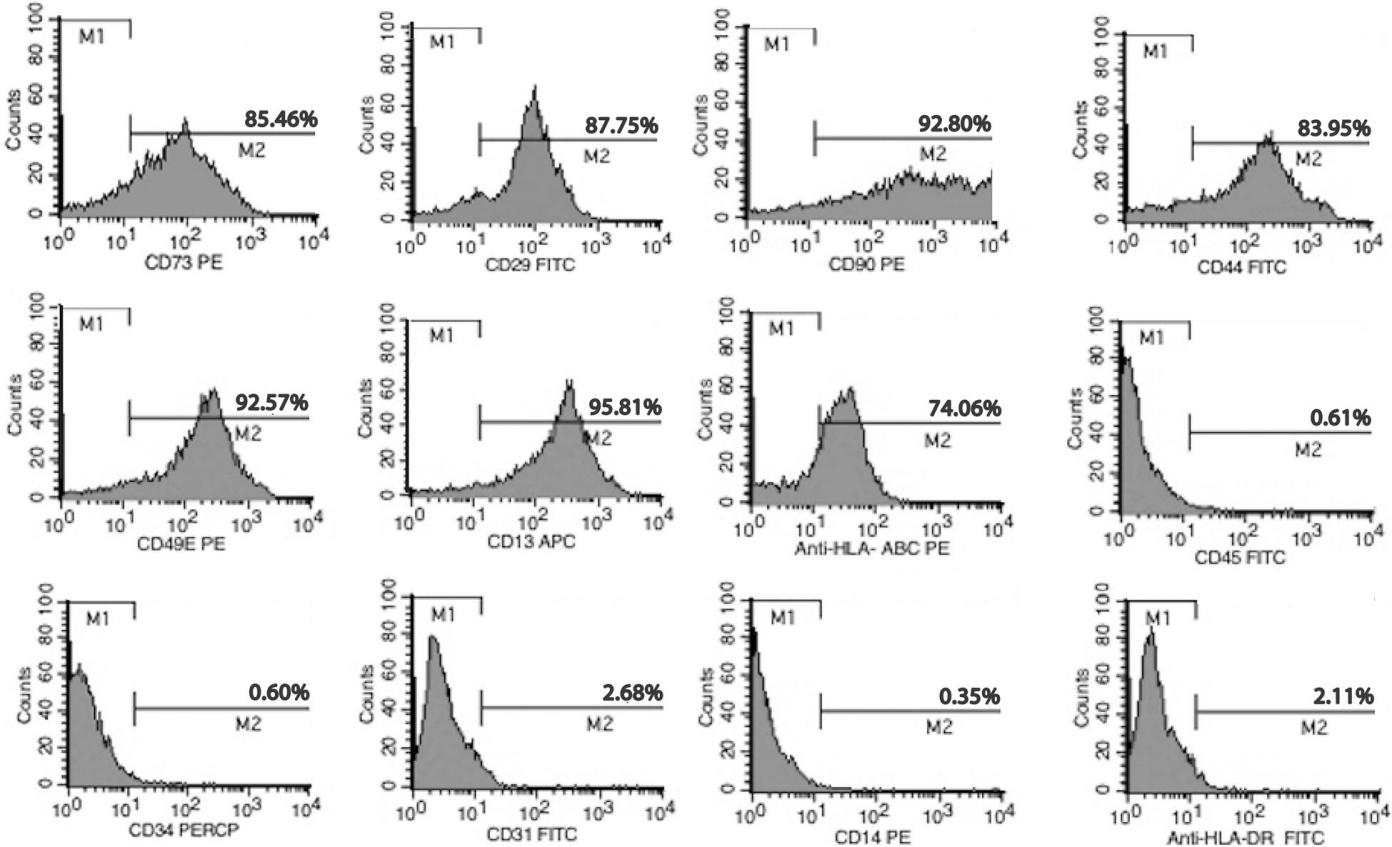
Characterization of pMSCs. Flow cytometry histograms show the expression levels of CD73, CD29, CD90, CD44, CD49e, CD13, HLA-ABC, CD45, CD34, CD31, CD14, and HLA-DR on pMSCs. Representative results from three independent experiments (biological replicates) are shown

Cultured pMSCs isolated from placental cotyledons showed capacity for differentiation into adipocytes and osteoblasts (data not shown). Furthermore, pMSCs presented typical MSC immunophenotype, with positive expression to CD13 (95.81%), CD29 (87.75%), CD44 (83.95%), CD49e (92.57%), CD73 (85.46%), CD90 (92.8%), and HLA-class I (74.06%) and lack of CD14 (0.35%), CD34 (0.6%), CD31 (2.68%), CD45 (0.61%), and HLA-class II (2.11%) markers (Fig. 1).

### LL-37 did not influence pMSC proliferation and viability

**Fig. 2:**
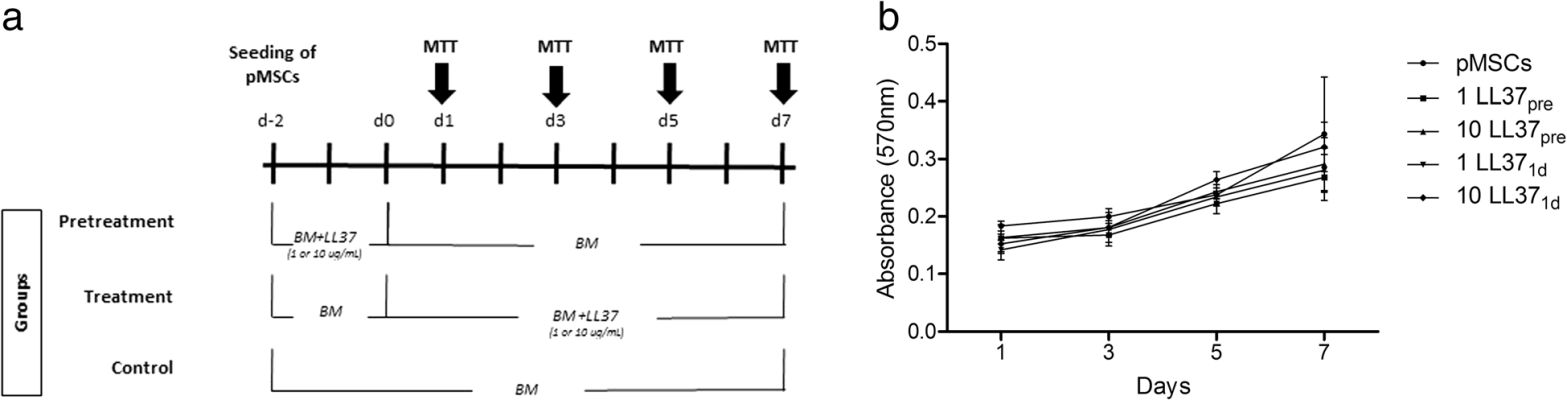
pMSCs proliferation and viability. **a** Experimental schedule. *BM* basal medium. **b** Untreated pMSCs, pMSCs pretreated with 1 ug/mL of LL-37 (1 LL37_pre_) or with 10 ug/mL of LL-37 (10 LL37_pre_), and pMSCs treated with 1 ug/mL LL-37 (1 LL37_1d_) or with 10 ug/mL of LL-37 (10 LL37_1d_) were cultured for 7 days, then cell proliferation/viability was assessed by MTT. There was no difference of proliferation/viability among groups. Values represent the means ± SEM. Three independent experiments (biological replicates) were performed. *LL-37* cathelicidin LL-3. *pMSCs* human placenta-derived MSCs

In order to assess the effects of LL-37 in pMSC proliferation and viability, we performed the MTT assay in untreated (control); pretreated with LL-37; or treated with LL-37 pMSCs (Fig. 2a). No changes were observed in pMSC viability or proliferation, regardless of the time of evaluation, treatment regimen and concentration of LL-37 performed (Fig. 2b).

### LL37 induces pMSCs migration

**Fig. 3:**
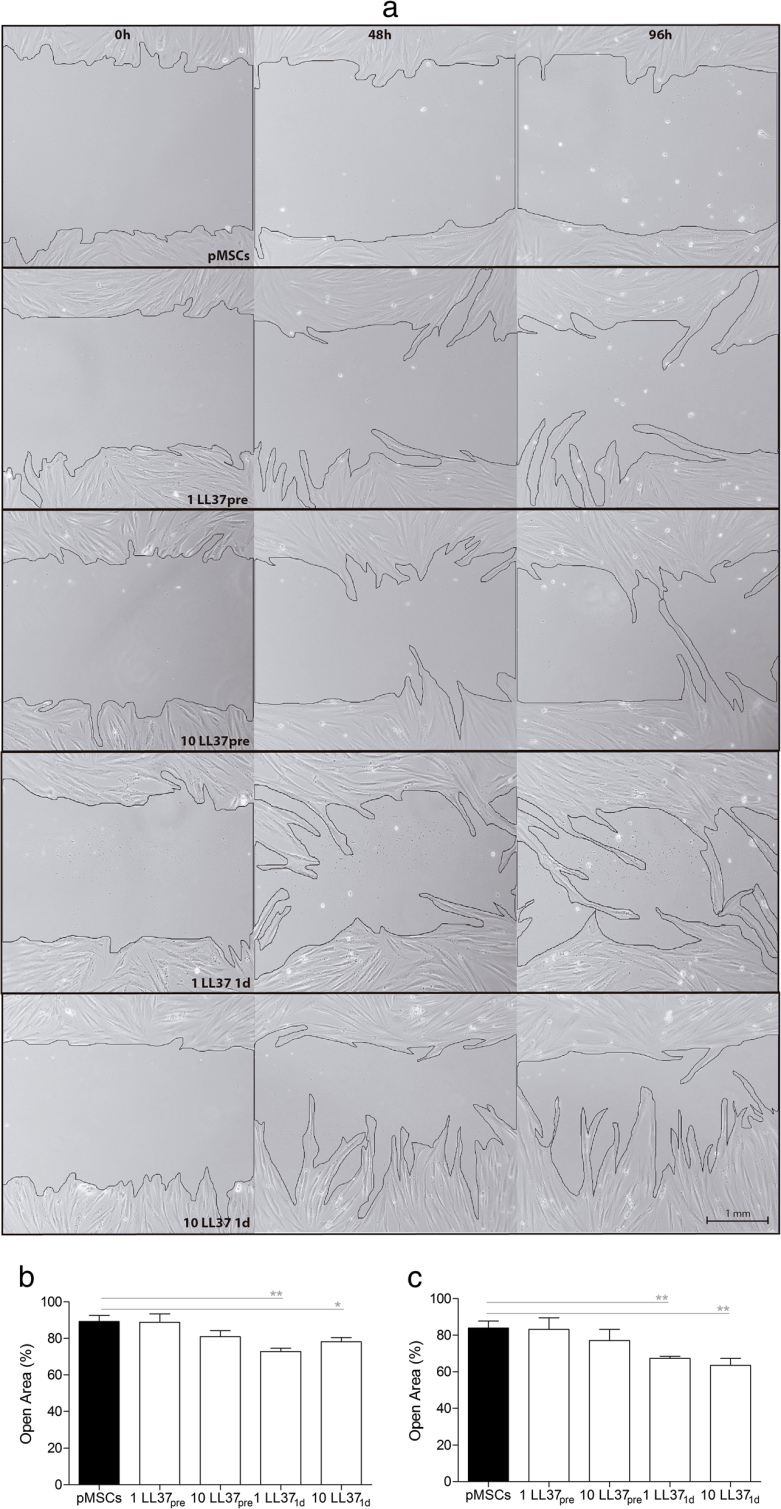
LL-37 induces pMSCs migration. **a** Wound scratch assay for untreated pMSCs; pretreated with LL-37; or treated with LL-37. Confluent cells in medium were wounded by a scratch with a pipette tip, and cell migration was monitored under the microscope. Representative results from three experiments (biological replicates) are shown. Quantitative analysis of wound-induced migration assay after 2 days (**b**) and 4 days (**c**). Results are presented as mean ± SEM of three experiments (biological replicates). *p <0.05. **p <0.01. *LL-37* cathelicidin LL-3. *pMSCs* human placenta-derived MSCs

The effects of LL-37 in pMSCs migration were investigated by wound scratch assay in untreated (control), pretreated with LL-37 or treated with LL-37 pMSCs (Fig. 3a). The pretreatment of pMSCs with 1 and 10 μg/mL of LL-37 did not influence the migratory cells ability. However, the treatment with 1 μg/mL of LL-37 enhanced the migratory potential of pMSCs after 48 h (*p* = 0.005) (Fig. 3b) and 96 h (*p* = 0.007) of culture (Fig. 3c). Likewise, an increased migratory behavior of pMSCs was detected in cells treated with 10 μg/mL of LL-37 for 48 (*p* = 0.02) and 96 h (*p* = 0.009).

### LL-37 enhances pMSCs immunomodulation

**Fig. 4:**
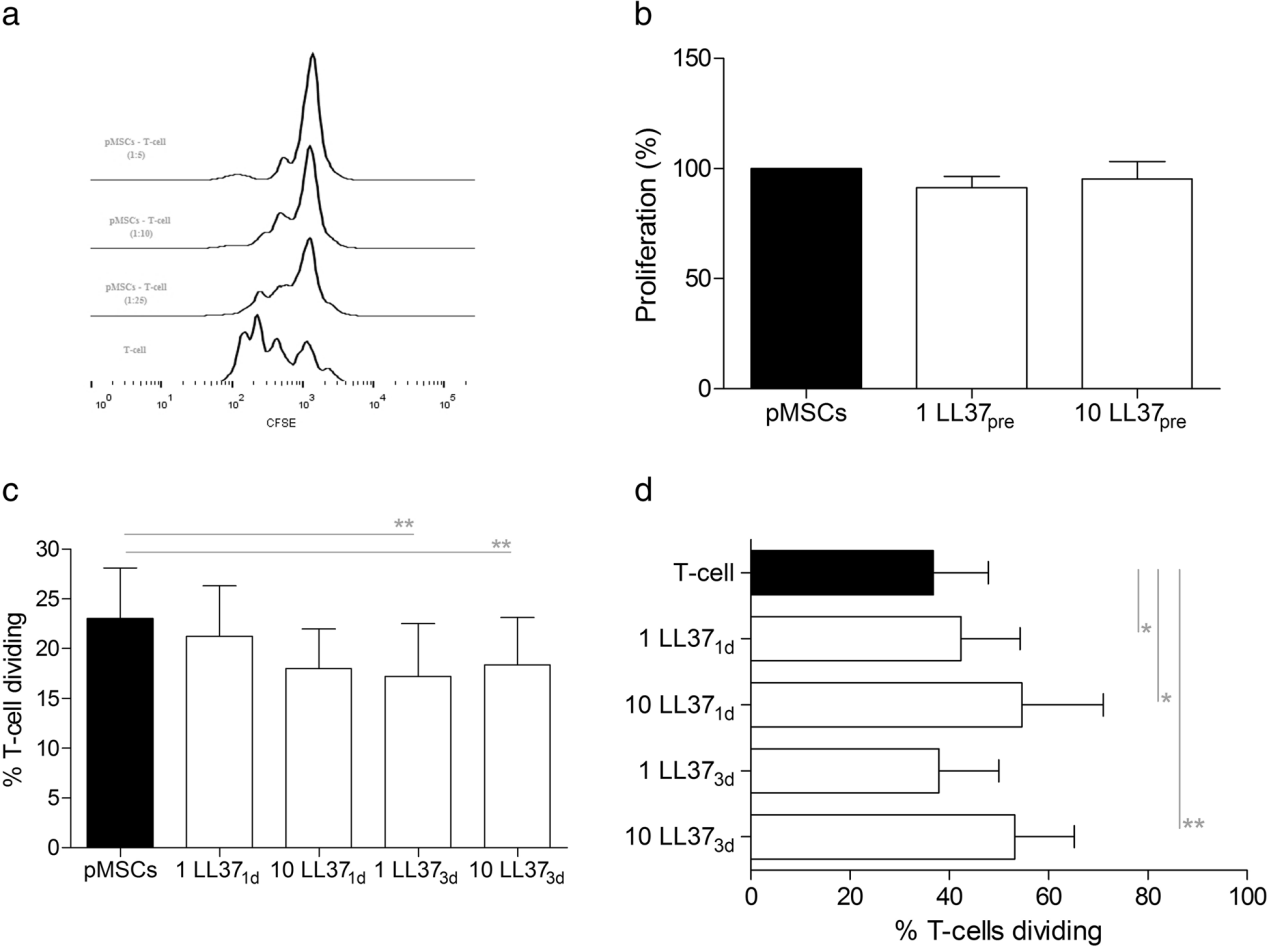
LL-37 enhances pMSCs immunomodulation. **a** pMSCs were co-cultured with PHA-activated PBMCs in different ratios and T cell proliferation determined for flow cytometry after 5 days. pMSCs inhibited T cell proliferative response in a dose-dependent manner. **b** The immunosuppressive capacity of pMSCs is not influenced by the pretreatment with LL-37 (1 LL37_pre_ and 10 LL37_pre_). **c** pMSCs were co-cultured for 5 days with PHA-activated PBMCs and LL-37 was added in the first (1 LL37_1d_ and 10 LL37_1d_) or in the third (1 LL37_3d_ and 10 LL37_3d_) day of culture. LL-37 enhanced the anti-proliferative effects of pMSCs on T cells. **d** PHA-activated PBMCs were cultured for 5 days, LL-37 being added in the first (1 LL37_1d_ and 10 LL37_1d_) or third (1 LL37_3d_ and 10 LL37_3d_) day of culture. LL-37 increased T cell proliferation. pMSCs and PBMCs from three healthy volunteers were used in the experiments. Results are presented as mean ± SEM. *p <0.05. **p <0.01. *LL-37* cathelicidin LL-3, pMSCs human placenta-derived MSCs

Proliferation of activated T cells was inhibited by pMSCs obtained from placental cotyledons in a dose-dependent manner (Fig. 4a). To evaluate the effects of LL-37 in this pMSCs property, we initially investigated if the pretreatment of pMSCs with LL-37 could influence the immunomodulatory function of these cells (Fig. 4b). Data indicates that the culture of pMSCs with LL-37 for 2 days did not modify the capacity of these cells to control T cell proliferation in the absence of LL-37. Another investigation made was if the LL-37 would act as an adjuvant and exert any effect over the pMSCs-PBMCs co-culture. Interestingly, when added on the third day of culture, regardless if at 1 or 10 μg/mL, LL-37 enhanced the anti-proliferative effects of pMSCs on T cells (*p* = 0.001 and *p* = 0.007, respectively). Although not statistically significant, the addition of 10 μg/mL LL-37 on the first day of the culture slightly reduced the proliferation of T cells in approximately 20% (*p* = 0.059), compared to untreated pMSCs (Fig. 4c). In order to investigate if the effect observed could be the result of direct immunosuppressive action of LL-37 on PBMCs, we added LL-37 in the culture of activated PBMCs alone. Surprisingly, the addition of 1 and 10 μg/mL LL-37 in the first day of culture in fact enhanced T cell proliferation (*p* = 0.01 and *p* = 0.04, respectively). Likewise, treatment of PBMCs with 10 μg/mL LL-37 in the third day of culture also increased T cell proliferation (*p* = 0.002) (Fig. 4d).

### LL-37 induces overexpression of TLR3 on pMSCs

**Fig. 5:**
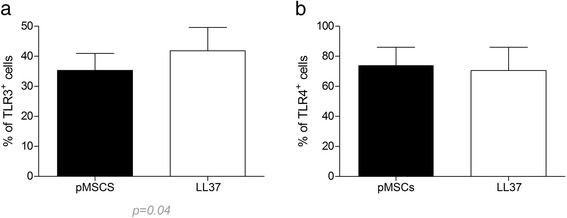
TLR3 and TLR4 expression on pMSCs. pMSCs were cultured in the presence or absence of LL-37 for 2 days. Expression of TLR3 (**a**) and TLR4 (**b**) was determined by flow cytometry. LL-37 induced TLR3 expression on pMSCs but had no effect on TLR4 expression. Results are presented as mean ± SEM of three experiments using three different pMSC donors. *LL-37* cathelicidin LL-3, 7, *pMSCs* human placenta-derived MSCs, *TLR* Toll-like receptor

In order to investigate a possible mechanism by which LL-37 boosted pMSC immunosuppressive behavior, pMSC expression of TLR3 and TLR4 were assessed by flow cytometry in pMSC treated with LL-37. Compared to untreated control, the peptide did not modify TLR4 expression on treated pMSCs, but induced an increased expression of TLR3 in these cells (*p* = 0.04) (Fig. 5).

### pMSCs express EGFR1 and produce anti-inflammatory factors in response to LL37 stimulation

**Fig. 6:**
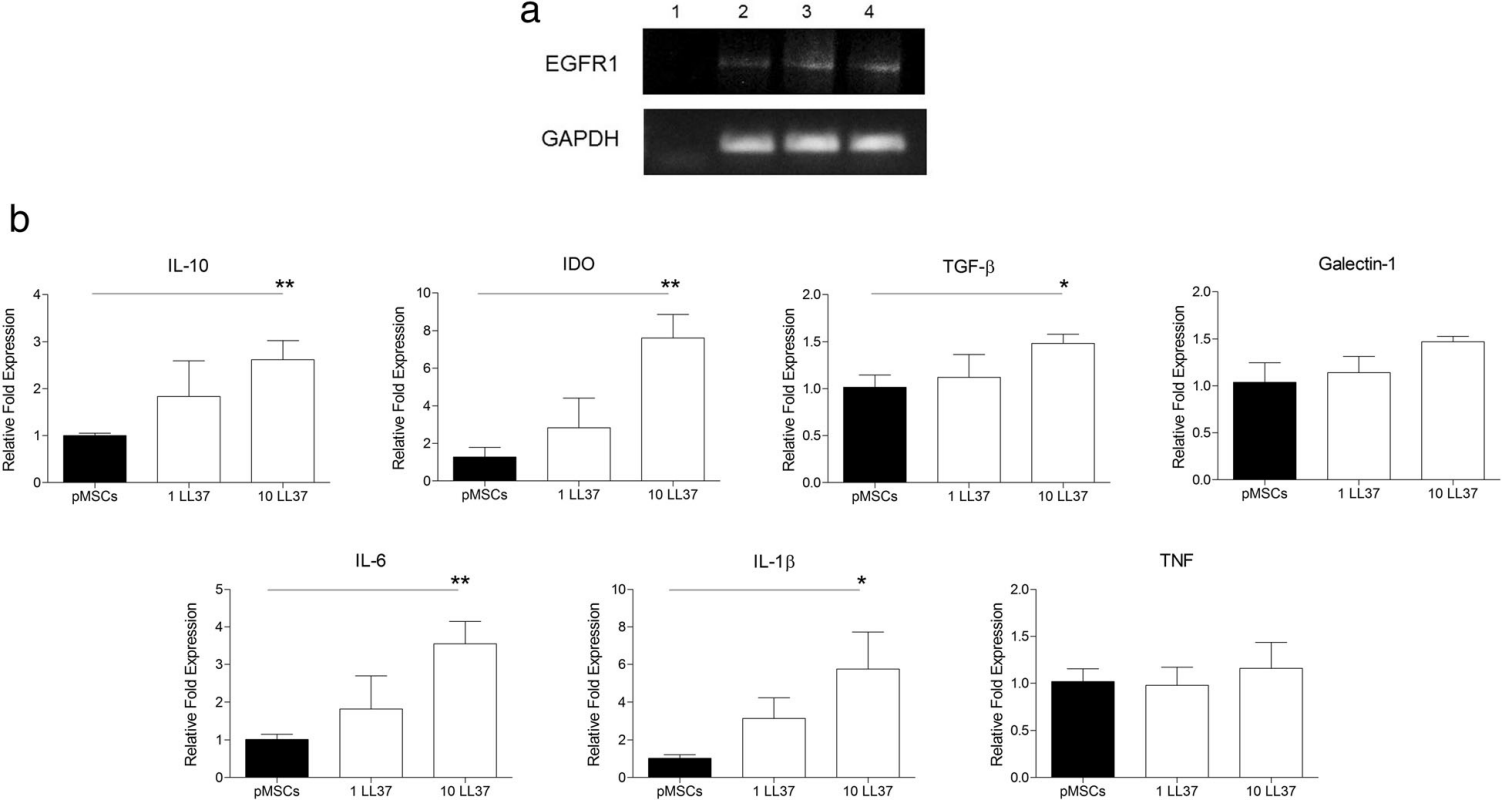
Gene expression analysis of selected transcripts. pMSCs were cultured in the presence or absence of LL-37 for 2 days, and profiled by real-time PCR according to (**a**) LL-37 receptors (EGFR and FPRL-1) and (**b**) pro-inflammatory and anti-inflammatory factors (IL-10, IDO, TGF-β, Galectin-1, IL-6, IL-1β, and TNF). The relative fold values were obtained by the 2 − ΔΔCt method, using the median Ct value of untreated MSCs as a reference. Results are presented as mean of three experiments (biological replicates). *p <0.05. **p <0.01*. EGFR1* epidermal growth factor receptor 1, *IDO* indoleamine 2,3-dioxygenase, *IL-10* interleukin 10, *IL-1*β*1* interleukin 1 beta, *IL-6* interleukin 6, *pMSCs* human placenta-derived MSCs, *TNF* tumor necrosis factor

Expression of EGFR1 was assessed in untreated pMSCs, as well as in pMSCs treated with 1 and 10 μg/mL of LL-37 for 2 days. Data shows that pMSCs express EGFR1 in all conditions (Fig. 6a). Real-time PCR revealed that transcript levels of IL-1β (*p* = 0.03), IL-6 (*p* = 0.007), IL-10 (*p* = 0.008), TGF-β (*p* = 0.02), and IDO (*p* = 0.004) were all significantly increased in pMSCs treated with 10 μg/mL of LL-37 (Fig. 6b). Although not statistically significant, the treatment of pMSCs with LL-37 increased the levels of galectin-1 transcript in 50% (*p* = 0.058), compared to untreated pMSCs. TNF expression was not disturbed by LL-37.

## Discussion

The present work shows that LL-37 does not compromise pMSC viability and proliferation, but increases their migratory behavior and boosts the capacity of these cells to control T cell proliferation following mitogenic stimulation. Furthermore, we demonstrated that LL-37 modulates TLR3 expression on pMSCs and induces high transcript levels of several anti-inflammatory factors that may compose some of the mechanisms by which MSCs suppress T cell responses. Our data builds on a conflicting literature, which reports contrasting effects of LL-37 regarding the protection or induction of cell apoptosis, depending on the cell type [26, 33]. Using 1 and 10 μg/mL of LL-37, we demonstrated that this peptide exerts no effect in pMSC viability. Likewise, Yang et al. [42], found no effect of LL-37 over MSC viability, but actually showed that LL-37 stimulates the proliferation of adipose-derived MSCs, under serum deprivation conditions. In order to simulate a physiological milieu, the effects of LL-37 were evaluated in culture medium supplemented with FBS, wherein this peptide did not affect the pMSCs proliferation. Considering that the immunosuppressive effect of MSCs is dose-dependent, the increase of MSCs proliferation could positively influence the cells’ immunoregulatory function. Nevertheless, we were able to detect such effect of LL-37 over pMSC immunoregulatory function, regardless of any detected proliferative role of this peptide.

Among the several functions of LL-37 in the immune response, this peptide may promote cell migration, as demonstrated on neutrophils [43] and mast cells [44]. This effect is of interest in the context of MSC therapy. In fact, the inefficient migratory capacity of MSCs is considered an important factor to be overcome in order to ensure the success in the clinical application of these cells, as discussed by [45–47]. Our data shown that pMSCs express EGFR, a receptor to LL-37 that controls MSCs migration [48, 49]. The demonstration that LL-37 acts on MSC migration has been revealed in a mice model of ovarian cancer, where LL-37 neutralization reduced the engraftment of MSCs in the tumor and lead to the inhibition of cancer progression [36]. In addition, it was shown that EGR1 [42] and TLR3 [50] are critical factors involved in MSC migration stimulated by LL-37. Interestingly, LL-37 is able to directly promote TLR3 signaling [51, 52] and this effect has been assessed in vitro by the detection of IL-6 [51, 53, 54]. Our findings are in line with these observations, being that pMSCs treatment with LL-37 enhanced the migratory capacity of these cells, induced high expression of TLR3 and increased IL-6 levels. However, it is important to emphasize that this effect appears to be dependent on the continuous LL-37 stimulus, since no pMSC migration effects were observed when these cells were pretreated with this peptide.

Besides contributing for MSC migration, it has been described that TLR3 is capable of polarizing MSCs toward an anti-inflammatory phenotype with enhanced immunosuppressive capacity [11, 12]. According to our data, LL-37 treatment of MSCs induced TLR3 signaling and boosted the capacity of these cells to control T cell proliferation. In order to dissect this effect, we investigated if this event could be derived from a direct suppressive effect of LL-37 on T cells. Surprisingly, the addition of this peptide to the culture of PHA-activated PBMCs enhanced T cell proliferation. It is important to emphasize the possibility that this pronounced effect of LL-37 on T cell proliferation may work as a double-edged sword, demanding a potent suppressive effect of pMSCs. In this sense, we demonstrated that LL-37 stimulates the overexpression of a broad arsenal of factors described to exert central effects on MSC-mediated immunomodulation, such as IL-10 [55], IL-1β [56], IDO [57], and TGF-β [58]. Once again, we underscore that the pronounced pMSC-suppressive effect over T cell proliferation was only seen when LL-37 was added directly to the co-culture system. Pretreatment of pMSCs with LL-37 was not sufficient to improve the suppressive potential of these cells on a longer observation period (5 days).

The literature reveals other important roles attributed to LL-37. For instance, this peptide appears to exert a protective function in MSCs against lipopolysaccharide (LPS) pro-inflammatory stimuli [50]. This observation is particularly relevant, given that LPS can bind to TLR4 on the surface of MSCs and polarize these cells to a pro-inflammatory phenotype, abolishing their potential in control T cell response in vitro and, more importantly, in vivo, which could explain, at least in part, why there are responders and non-responders in MSC therapies [12].

## Conclusions

Our data demonstrates that LL-37 modulates TLR3 expression, promotes higher levels of anti-inflammatory factors, and boosts the suppressive function of pMSCs over stimulated T cells. The positive results constitute important proof of concept, which stimulate further studies in the in vivo scenario, in order to investigate the effects of LL-37 in this complex and more realistic context. These results may also be of great relevance and open the possibility for a new therapeutic strategy for a highly efficient MSC-based therapy. More interestingly, in addition to boosting the immunossupressive and migratory potential of pMSCs, LL-37 may offer protection against opportunist microorganisms, ensuring the maintenance of MSCs in their highest anti-inflammatory state.

## Abbreviations

cDNA: complementary DNA
CFSE: carboxyfluorescein succinimidyl ester
EGFR: epidermal growth factor receptor
EGR1: early growth response 1
FBS: fetal bovine serum
FPRL1: formyl peptide receptor-like 1
GVHD: graft-versus-host disease
HDPs: host defense peptide
HPLC: high-performance liquid chromatography
IDO: indoleamine 2,3-dioxygenase
IL-10: interleukin 10
IL-1β: interleukin 1 beta
IL-6: interleukin 6
INF-ɣ: interferon gamma
LL-37: cathelicidin LL-37
LPS: lipopolysaccharide
MSCs: mesenchymal stem cells
MTT: 3-(4.5-dimethylthiazol-2-yl)-2,5-diphenyl tetrazolium bromide
P2X7: purinergic receptor P2X7
PBMCs: peripheral blood mononuclear cells
PBS: phosphate-buffered saline
PHA: phytohaemagglutinin
pMSC: human placenta-derived MSCs
RNA: ribonucleic acid
RPMI: Roswell Park Memorial Institute
TGF-β: transforming growth factor beta
TLR: Toll-like receptor
TNF: tumor necrosis factor
α-MEM: alpha-minimum essential medium
